# Supplementary material for: Two Onnamide Analogs from the Marine Sponge Theonella conica: Evaluation of Geometric Effects in the Polyene Systems on Biological Activity
Source: Molecules. 2023 Mar 9;28(6):2524. doi: 10.3390/molecules28062524 (PMC10058928; doi:10.3390/molecules28062524)
Supplement: Supplementary file 1 [file molecules-28-02524-s001.zip › molecules-2279444-supplementary.pdf]

Supporting Information

Two onnamide analogs from the marine sponge *Theonella conica*: evaluation of geometric effects in the polyene systems on biological activity

Fumiaki Nakamura [1], Hiroshi Kimura [2], Nobuhiro Fusetani [3], and Yoichi Nakao [1,3,*]

[1] Department of Chemistry and Biochemistry, Graduate School of Advanced Science and Engineering, Waseda University, 3-4-1 Okubo, Shinjuku-ku, Tokyo 169-8555, Japan
[2] Cell Biology Center, Institute of Innovative Research, Tokyo Institute of Technology, 4259 Nagatsuta, Midori-ku, Yokohama, 226-8501, Japan.
[*] Correspondence: ayocha@waseda.jp; Tel.: +81-3-5286-3100

Table of contents

Table S1. ^{1}H NMR spectral data [δ_H mult. (J in Hz)] for onnamides (**3**-**6**) in CD_3OD (400 MHz).

Table S2. The integrated value of ^{1}H NMR signal derived from compounds **1** to **4** when **3** was placed in an NMR tube and each time passed.

Figure S1. ESI spectrum (pos.) of 2*Z*-onnamide A (**1**).

Figure S2-1. ^{1}H NMR spectrum of 2*Z*-onnamide A (**1**) in MeOD.

Figure S2-2. ^{1}H NMR spectrum of 2*Z*-onnamide A (**1**) in MeOD (5.6-7.6 ppm).

Figure S3. ^{13}C NMR spectrum of 2*Z*-onnamide A (**1**) in MeOD.

Figure S4. COSY spectrum of 2*Z*-onnamide A (**1**) in MeOD.

Figure S5. HMQC spectrum of 2*Z*-onnamide A (**1**) in MeOD.

Figure S6. HMBC spectrum of 2*Z*-onnamide A (**1**) in MeOD.

Figure S7. ESI spectrum (pos.) of 6*Z*-onnamide A (**2**) in MeOD.

Figure S8-1. ^{1}H NMR spectrum of 6*Z*-onnamide A (**2**) in MeOD.

Figure S8-2. ^{1}H NMR spectrum of 6*Z*-onnamide A (**2**) in MeOD (5.6-7.4 ppm).

Figure S9. ^{13}C NMR spectrum of 6*Z*-onnamide A (**2**) in MeOD.

Figure S10. COSY spectrum of 6*Z*-onnamide A (**2**) in MeOD.

Figure S11. HMQC spectrum of 6*Z*-onnamide A (**2**) in MeOD.

Figure S12. HMBC spectrum of 6*Z*-onnamide A (**2**) in MeOD.

Figure S13. ^{1}H NMR spectrum of onnamide A (**3**) in MeOD.

Figure S14. ^{1}H NMR spectrum of 4*Z*-onnamide A (**4**) in MeOD.

Figure S15. ^{1}H NMR spectrum of dihydroonnamide A (**5**) in MeOD.

Figure S16. ^{1}H NMR spectrum of onnamide B (**6**) in MeOD.

Figure S17. ^{1}H NMR spectrum of onnamide A (**3**) over time in MeOD.

Figure S18. Effects of onnamide A (**3**) on 16 types of histone modifications.

Figure S19. The levels of H3K4me3, H3K27me3, H3K36me3, and H4K5ac after onnamides (**1**-**6**) treatment in histone modification assay**.**

Figure S20. The cell numbers after onnamides (**1**-**6**) treatment in histone modification assay.

Figure S21. The levels of H3K4me3, H3K27me3, H3K36me3, and H4K5ac after onnamide A (**3**) and anisomycin treatment in histone modification assay**.**

Table S1 ^{1}H NMR spectral data [δ_H mult. (J in Hz)] for onnamides (**3**-**6**) in CD_3OD (400 MHz).

position	onnamide A (**3**)	4*Z*-onnamide A (**4**)	dyhidroonnamide A (**5**)	onnamide B (**6**)
1				
2	6.07 d (15.0)	6.08 d (14.9)	6.01 d (15.1)	6.02 d (15.1)
3	7.14 dd (15.0, 11.2)	7.67 dd (14.9, 12.0)	7.11 dd (15.1, 10.7)	7.12 dd (15.1, 10.8)
4	6.26 dd (14.9, 11.2)	6.00 dd (12.0, 11.2)	6.21 dd (15.1, 10.7)	6.26 dd (15.2, 10.8)
5	6.52 dd (14.9, 10.7)	6.28 t (11.2)	6.10 dt (15.1, 6.8)	6.12 dt (15.2, 6.9)
6	6.22 dd (15.1, 10.7)	6.72 dd (14.9, 11.2)	2.19 q (6.0)	-
7	5.95 dt (15.1, 6.9)	5.97 dt (14.9, 7.3)	1.46 m	-
8	2.22 m, 2.13 m	2.19 m	1.31 m, 1.35 m	2.22 m, 2.16 m
9	1.56 m, 1.42 m	1.63 m, 1.48 m	1.31 m, 1.46 m	1.54 m, 1.44 m
10	1.31 m,1.50 m	1.30 m, 1.49 m	1.31 m,1.46 m	1.27 m,1.50 m
11	3.66 m	3.66 m	3.66 m	3.66 m
12	1.53 m	1.54 m	1.54 brt (6.1)	1.53 m
13	3.48 dd (8.4, 3.9)	3.48 dd (8.9, 3.0)	3.49 t (6.8)	3.48 dd (8.8, 3.6)
14				
15	3.64 d (9.8)	3.67 brd (9.9)	3.65 d (9.4)	3.65 d (9.9)
16	4.17 dd (9.8, 6.6)	4.17 dd (9.9, 6.5)	4.17 dd (9.8, 6.5)	4.17 dd (9.9, 6.5)
17	3.99 dd (9.3, 6.6)	4.00 dd (9.4, 6.5)	3.98 dd (9.3, 6.5)	3.98 dd (9.3, 6.5)
18	5.80 d (9.3)	5.81 d (9.4)	5.81 d (9.3)	5.81 d (9.3)
20				
21	4.24 s	4.24 s	4.24 s	4.24 s
22				
23	2.41 brd (14.3), 2.32 brd (14.3)	2.40 brd (14.0), 2.31 brd (14.0)	2.42 d (14.3), 2.33 d (14.3)	2.41 brd (14.3), 2.32 brd (14.3)
24				
25	2.19 m	2.19 m	2.20 m	2.19 m
26	3.88 qd (6.5, 2.6)	3.86 qd (6.5, 2.5)	3.88 qd (6.5, 2.6)	3.88 qd (6.5, 2.5)
27	1.18 d (6.5)	1.17 d (6.5)	1.18 d (6.5)	1.18 d (6.5)
28	0.97 d (7.0)	0.95 d (7.0)	0.97 d (7.0)	0.97 d (7.0)
29	4.79 brs, 4.65 brs	4.79 brs, 4.64 brs	4.79 brs, 4.65 brs	4.79 brs, 4.64 brs
30	3.24 s	3.21 s	3.25 s	3.24 s
31	5.20d (6.9), 4.80 d (6.9)	5.22 d (6.9), 4.79 d (6.9)	5.21 d (6.9), 4.80 d (6.9)	5.21d (6.9), 4.80 d (6.9)
32	3.56 s	3.56 s	3.56 s	3.56 s
33	0.86 s	0.86 s	0.87 s	0.86 s
34	1.01 s	1.01 s	1.01 s	1.00 s
1'				
2'	4.37 dd (7.9, 5.3)	4.40 dd (7.5, 5.0)	4.37 dd (7.4, 5.2)	4.37 dd (7.8, 5.3)
3'	1.90 m, 1.75 m	1.91 m, 1.75 m	1.90 m, 1.74 m	1.87 m, 1.73 m
4'	1.62 m	1.63 m	1.62 m	1.62 q (9.5)
5'	3.19 m	3.22 m	3.22 m, 3.17 m	3.22 m, 3.16 m
7'				

Table S2 The integrated value of ^{1}H NMR signal derived from compounds **1**-**4** when **3** was placed in an NMR tube and each time passed (the integrated value of H-3 in **3** is set to "1")

compound/position	time (hour)							
	0	24	48	72	96	168	672	840
onnamide A (**3**)/H-3	1	1	1	1	1	1	1	1
2*Z*-onnamide A (**1**) /H-4	0.03	0.03	0.03	0.05	0.05	0.06	0.09	0.12
4*Z*-onnamide A (**2**) /H-3	0.02	0.02	0.02	0.02	0.04	0.04	0.08	0.13
6*Z*-onnamide A (**4**) /H-3	n.d.	n.d.	n.d.	n.d.	n.d.	0.02	0.05	0.08

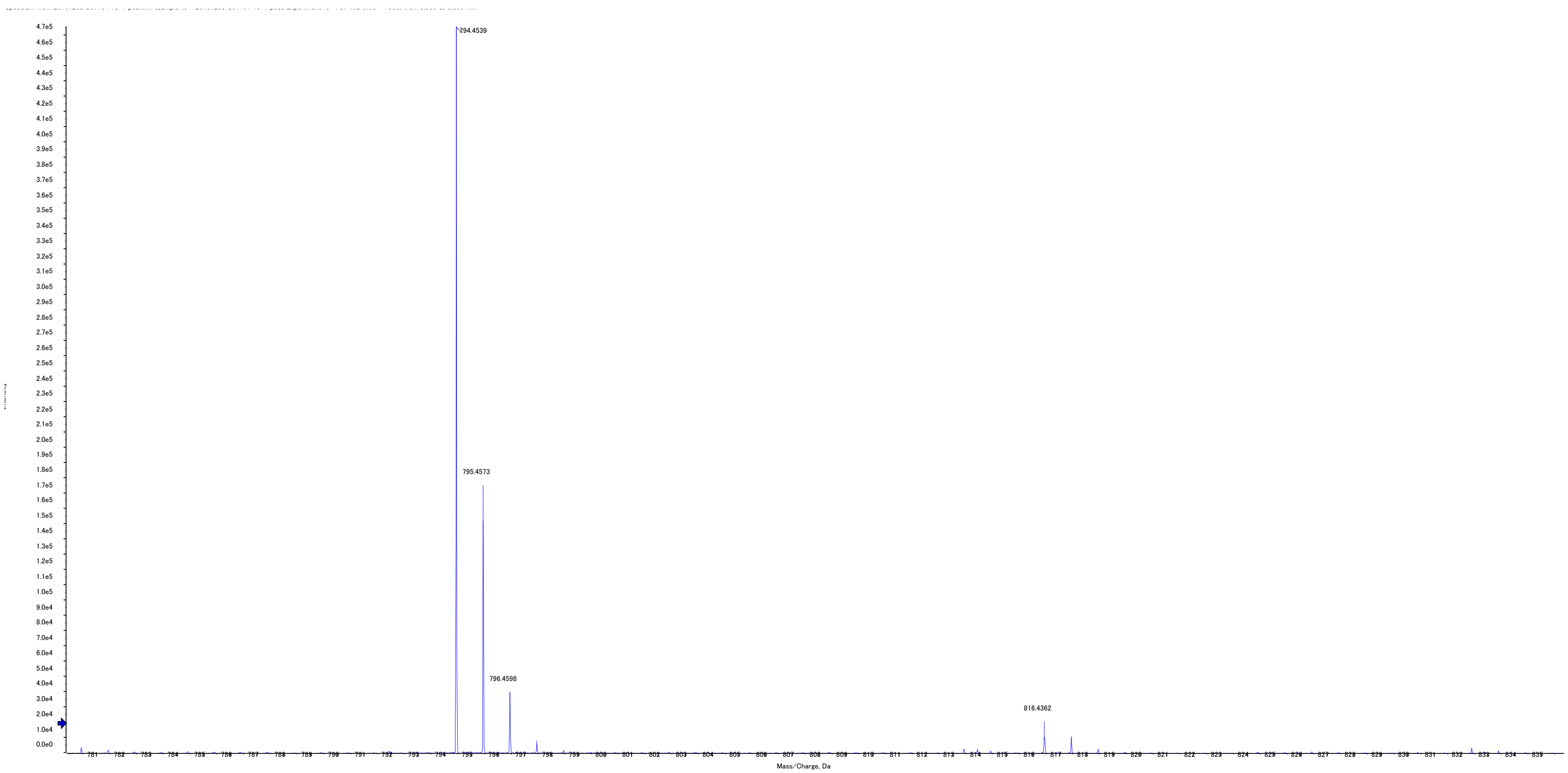

Figure S1. ESI spectrum of 2*Z*-onnamide A (**1**) in MeOD.

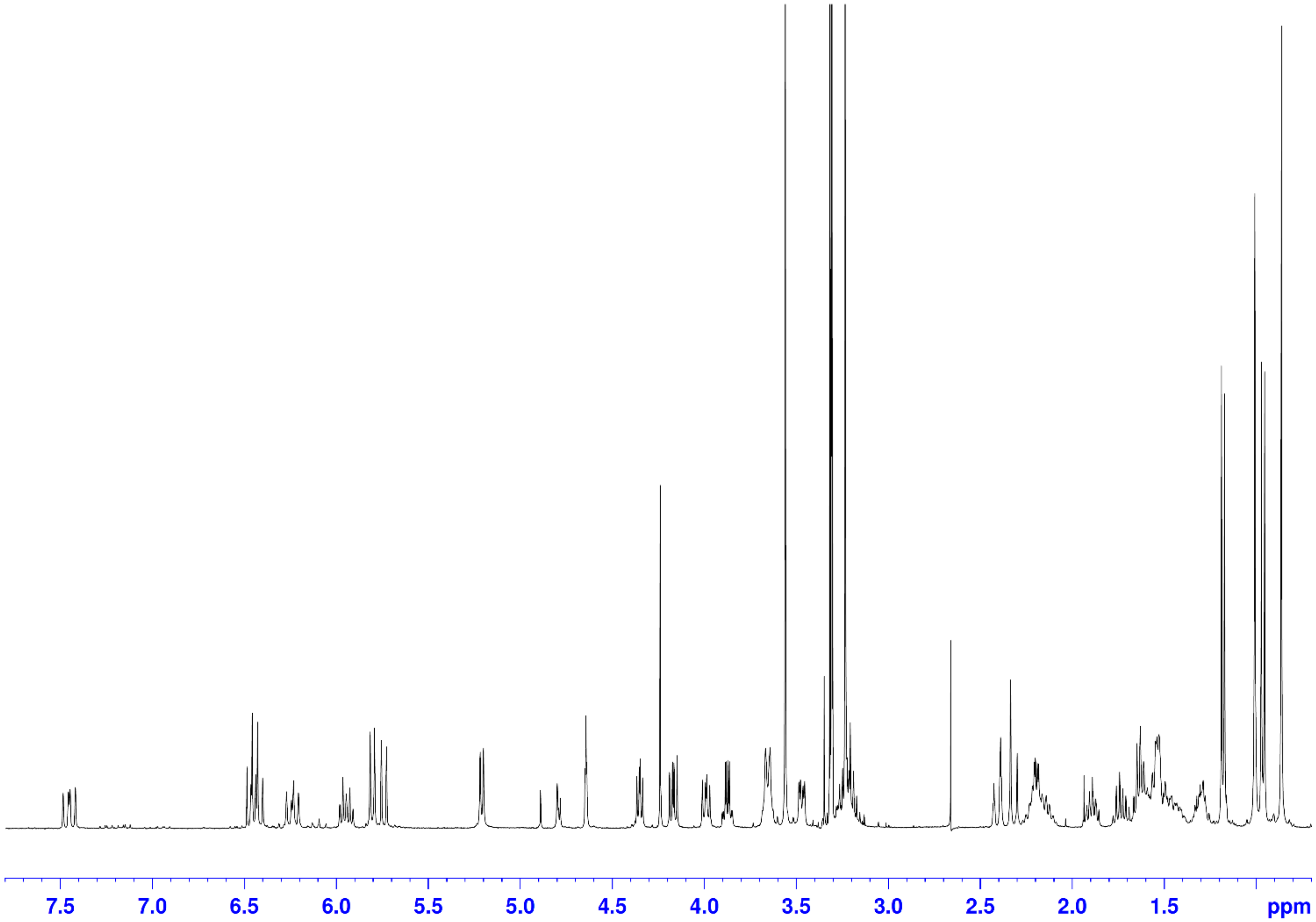

Figure S2-1. ^{1}H NMR spectrum of 2*Z*-onnamide A (**1**) in MeOD.

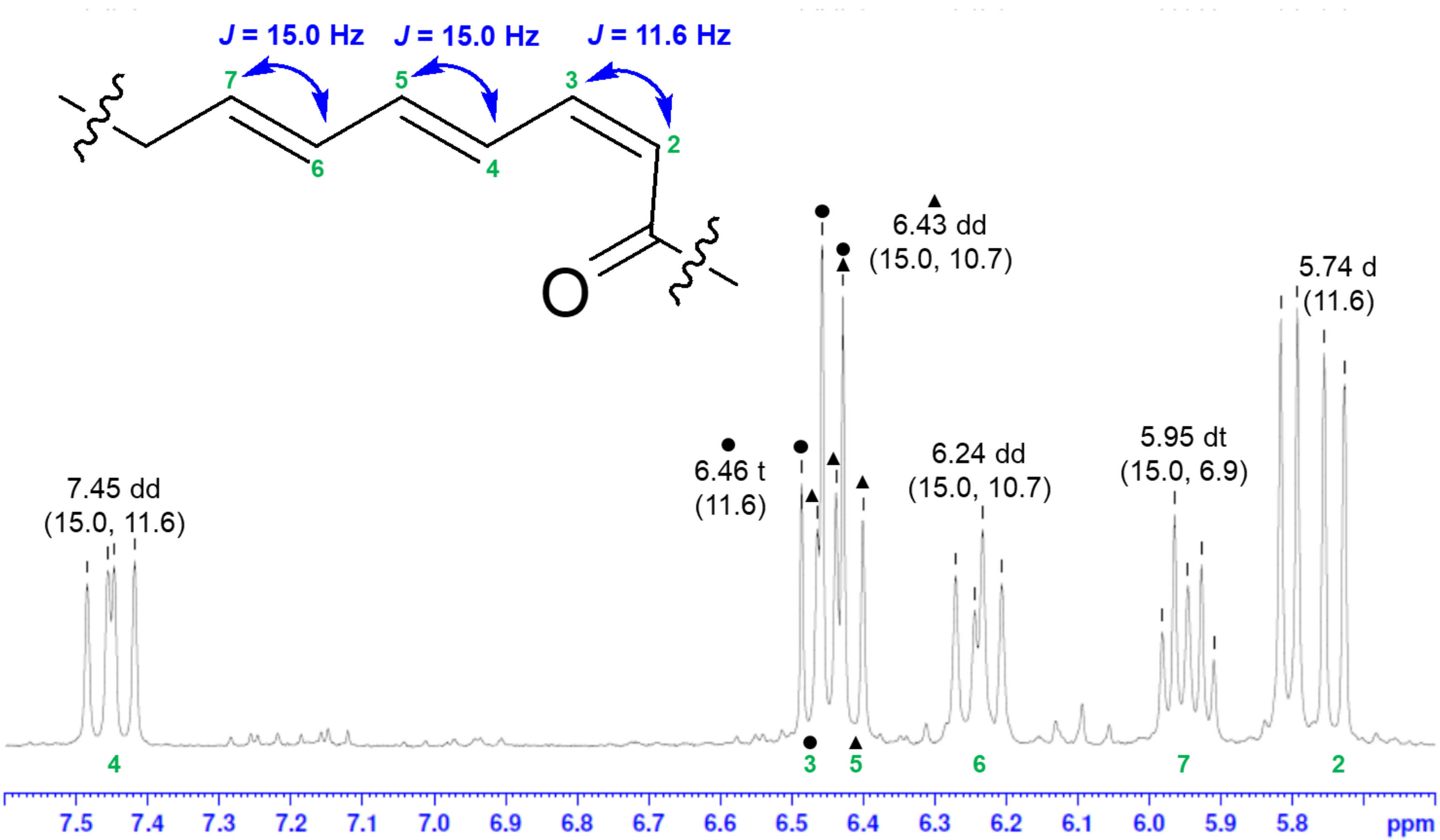

Figure S2-2. ^{1}H NMR spectrum of 2*Z*-onnamide A (**1**) in MeOD (5.6-7.6 ppm).

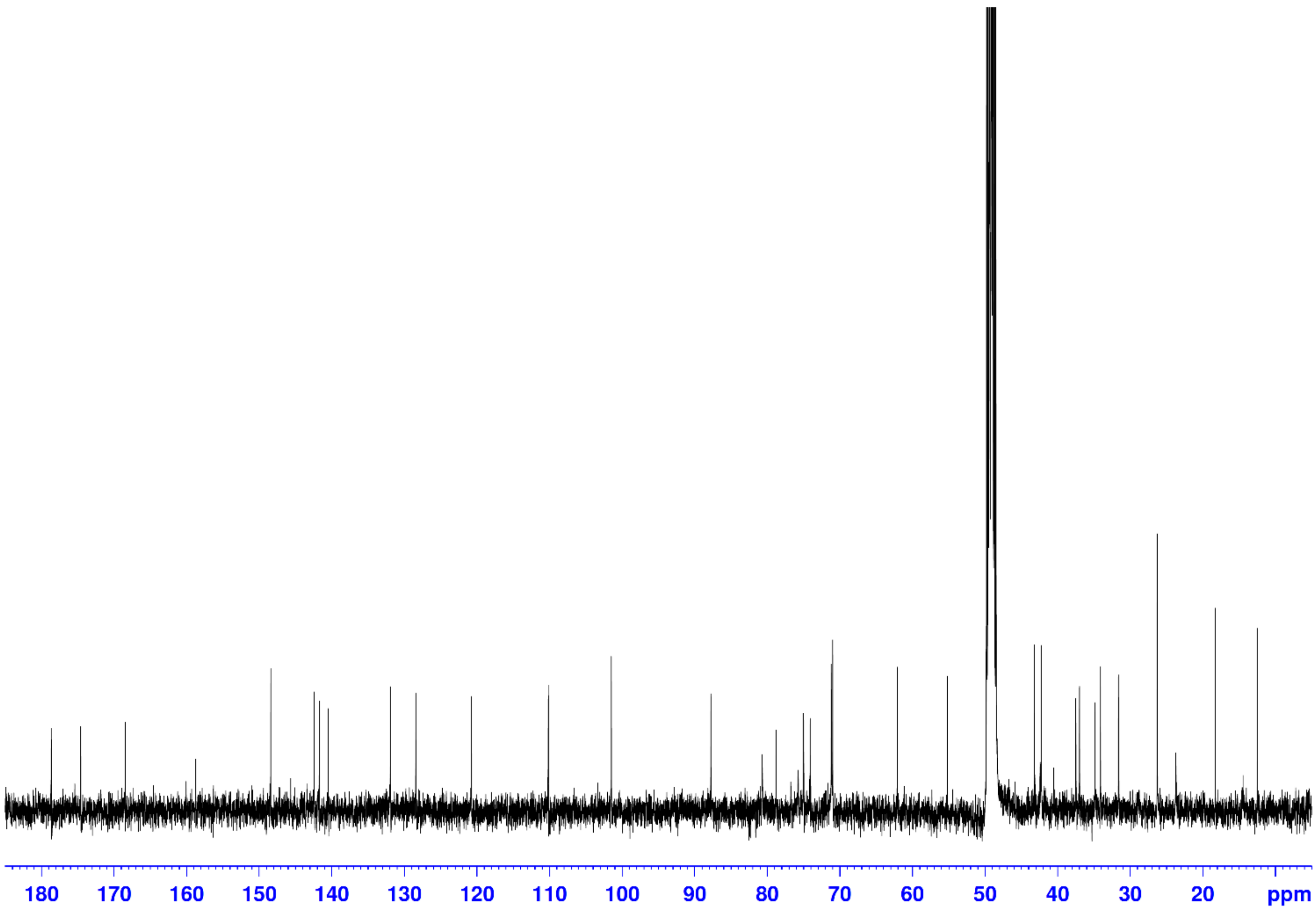

Figure S3. ^{13}C NMR spectrum of 2Z-onnamide A (**1**) in MeOD.

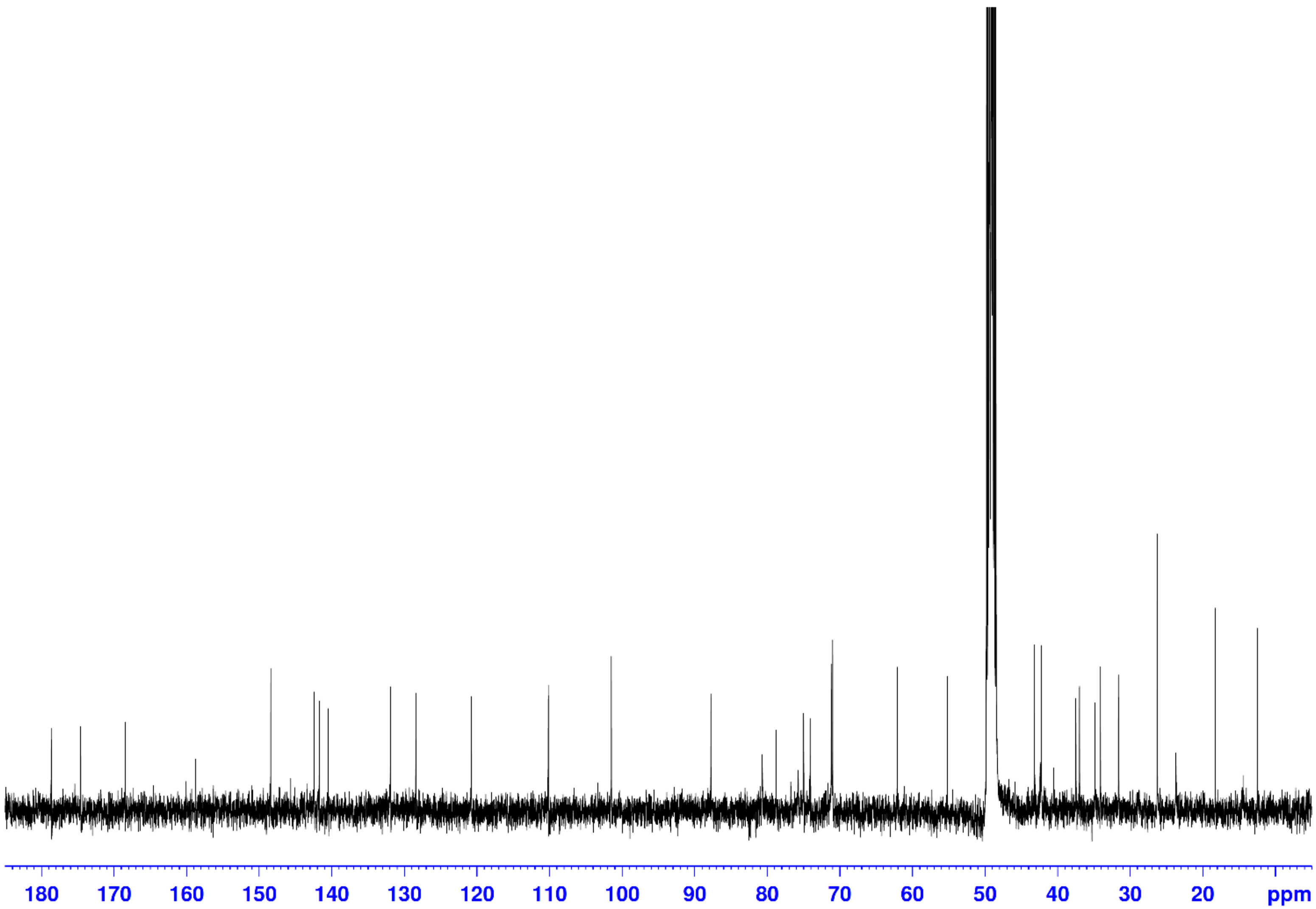

Figure S4. COSY spectrum of 2*Z*-onnamide A (**1**) in MeOD.

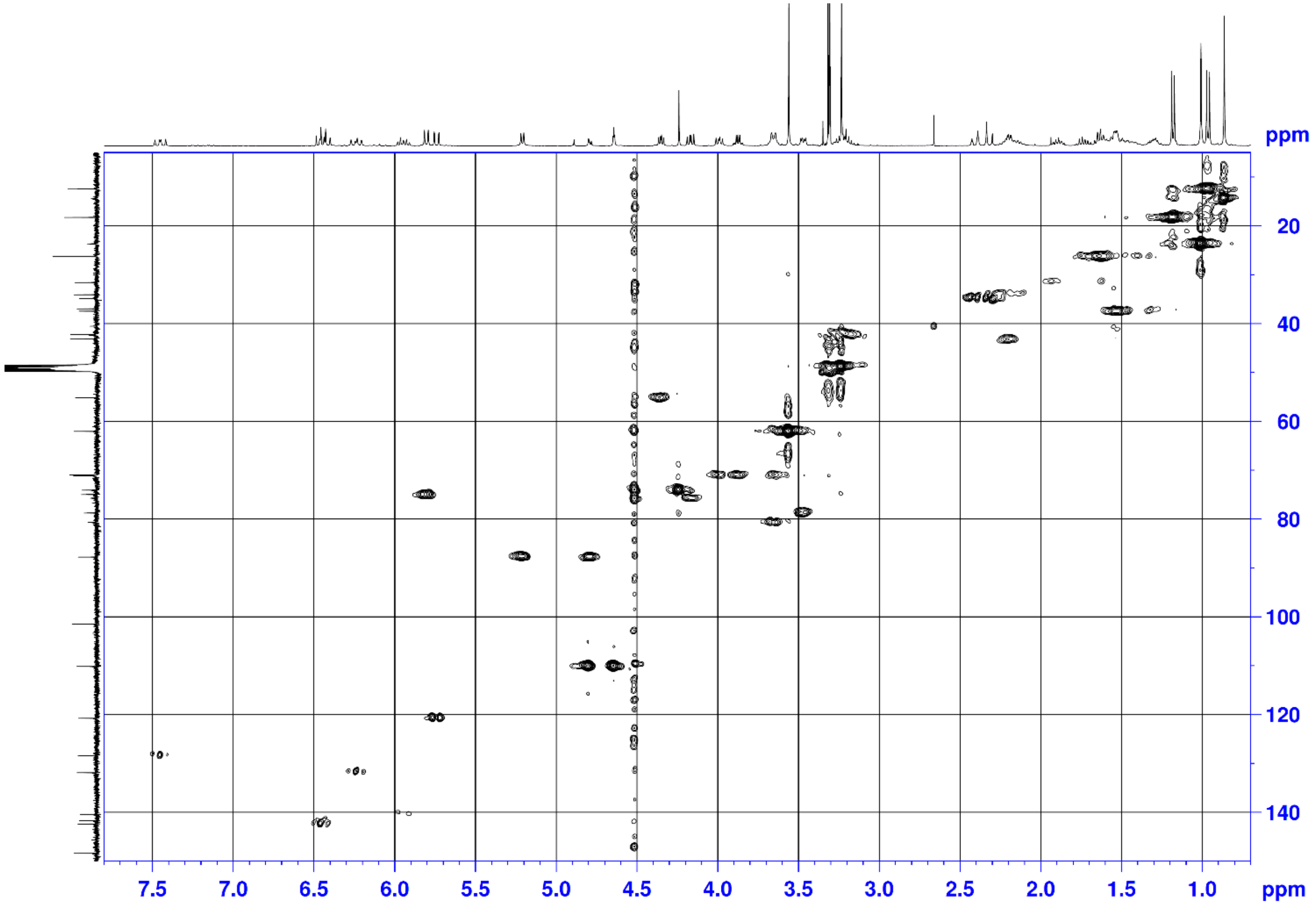

Figure S5. HMQC spectrum of 2Z-onnamide A (**1**) in MeOD.

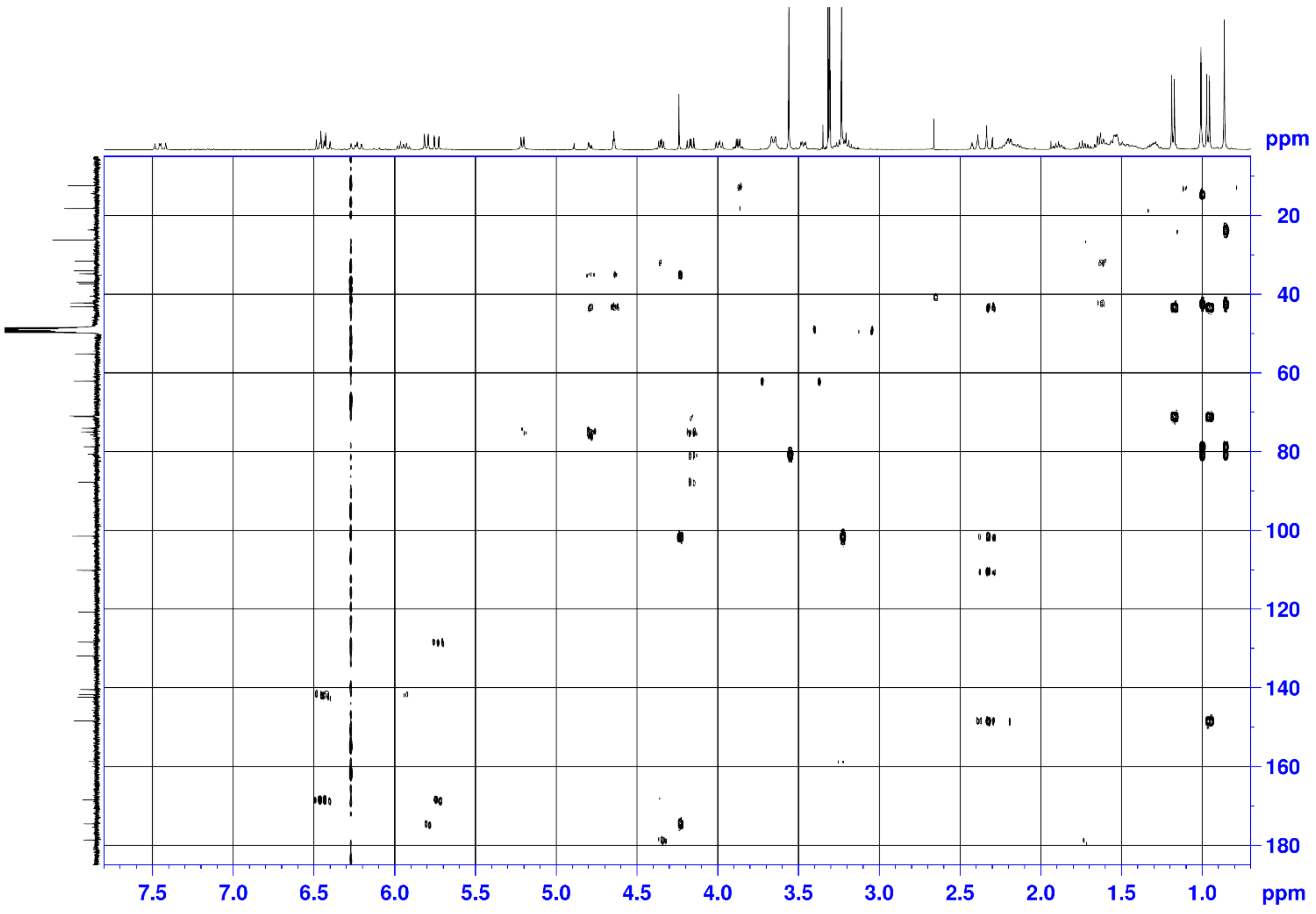

Figure S6. HMBC spectrum of 2Z-onnamide A (**1**) in MeOD.

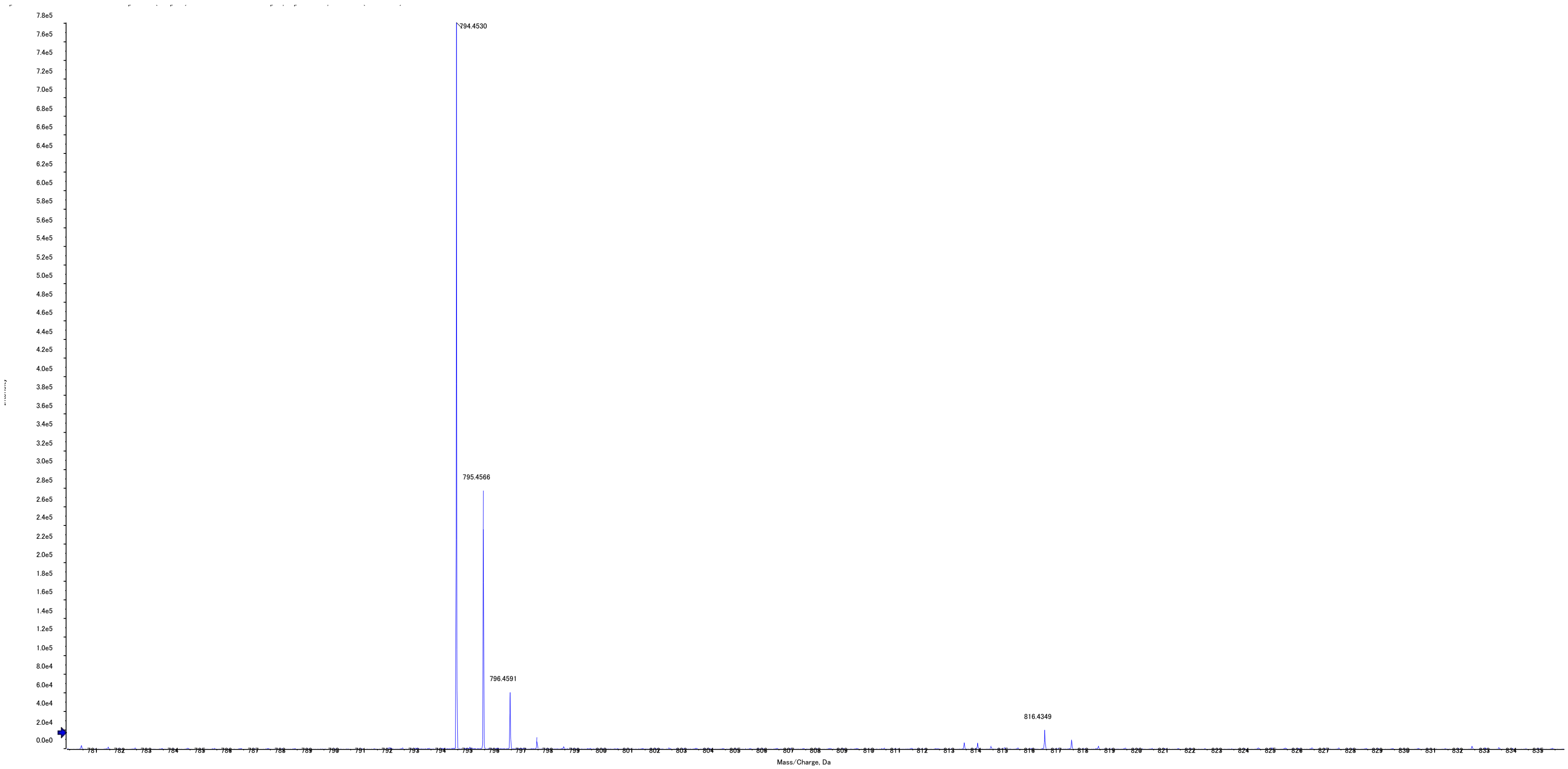

Figure S7. ESI spectrum (pos.) of 6Z-onnamide A (**2**).

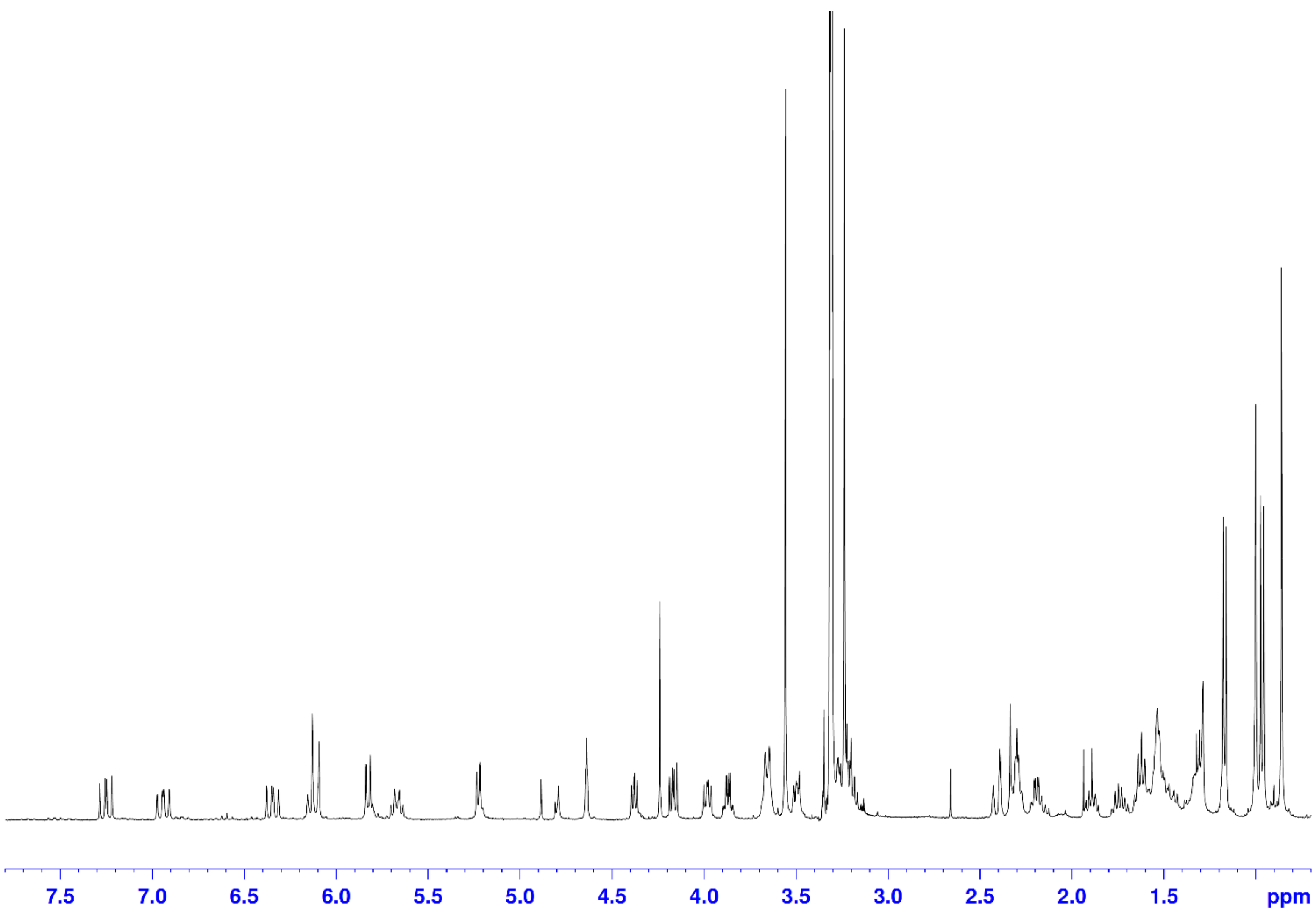

Figure S8-1. ^{1}H NMR spectrum of 6*Z*-onnamide A (**2**) in MeOD.

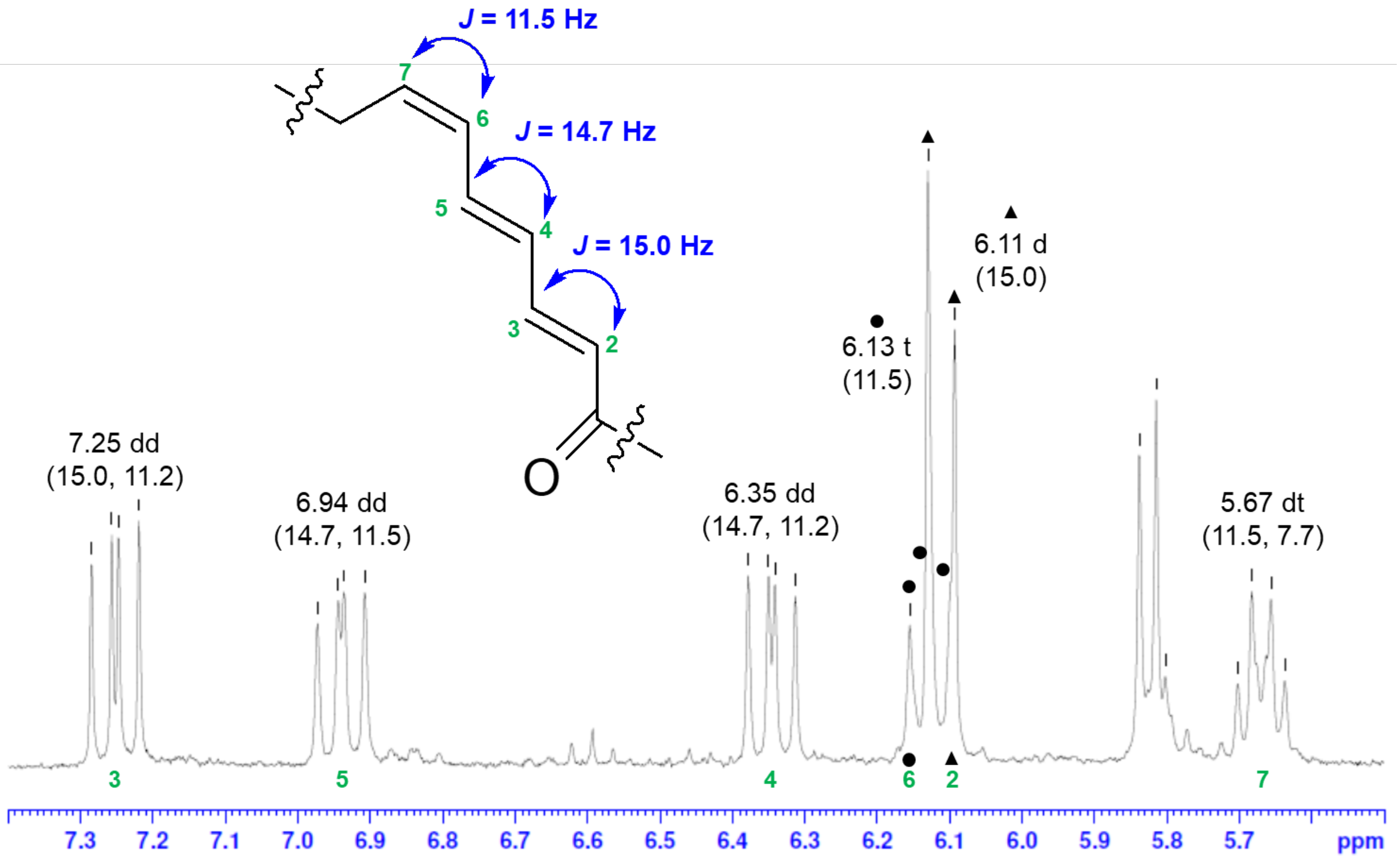

Figure S8-2. ^{1}H NMR spectrum of 6Z-onnamide A (**2**) in MeOD (5.6-7.4 ppm).

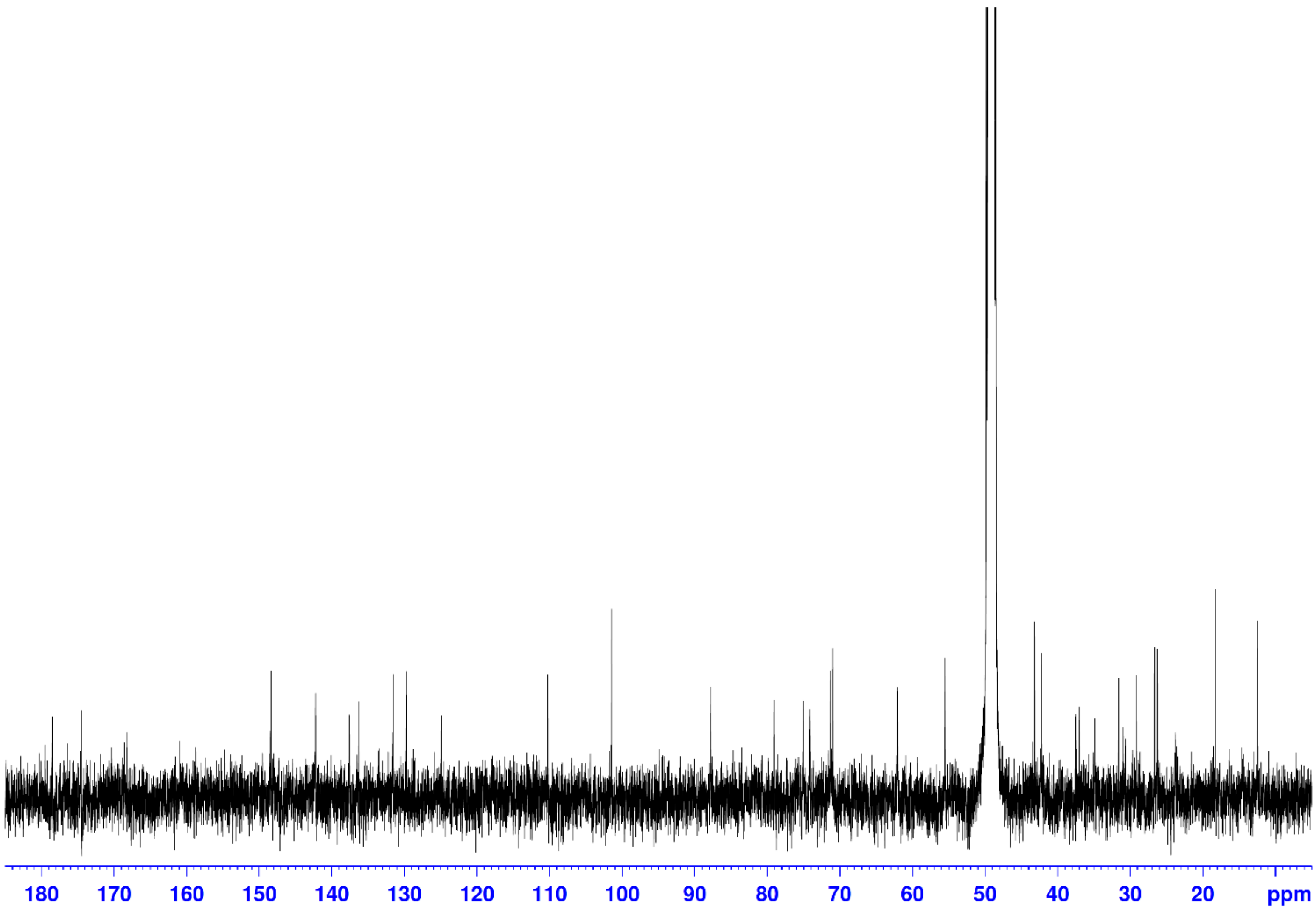

Figure S9. ^{13}C NMR spectrum of 6Z-onnamide A (**2**) in MeOD.

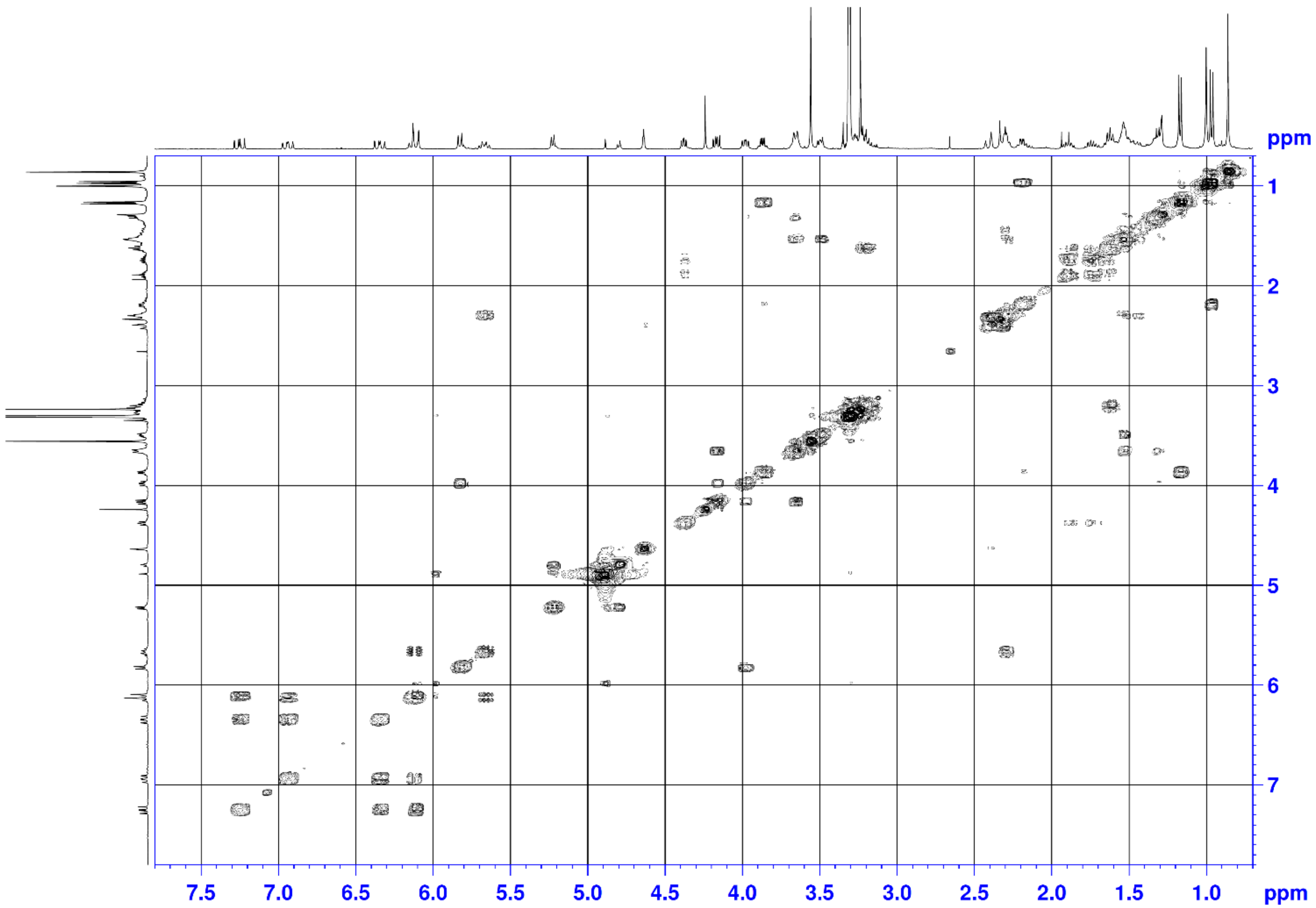

Figure S10. COSY spectrum of 6Z-onnamide A (**2**) in MeOD.

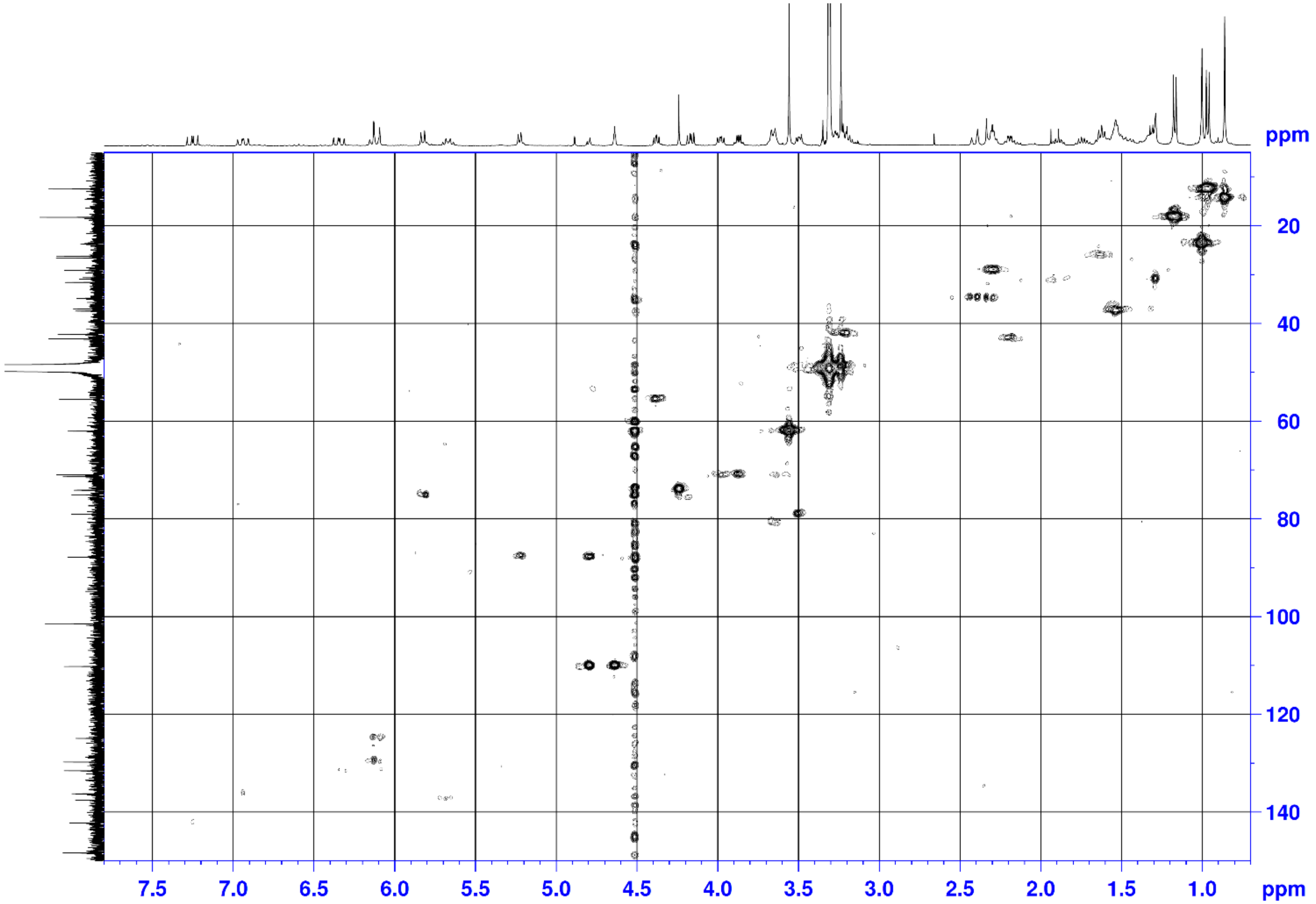

Figure S11. HMQC spectrum of 6Z-onnamide A (**2**) in MeOD.

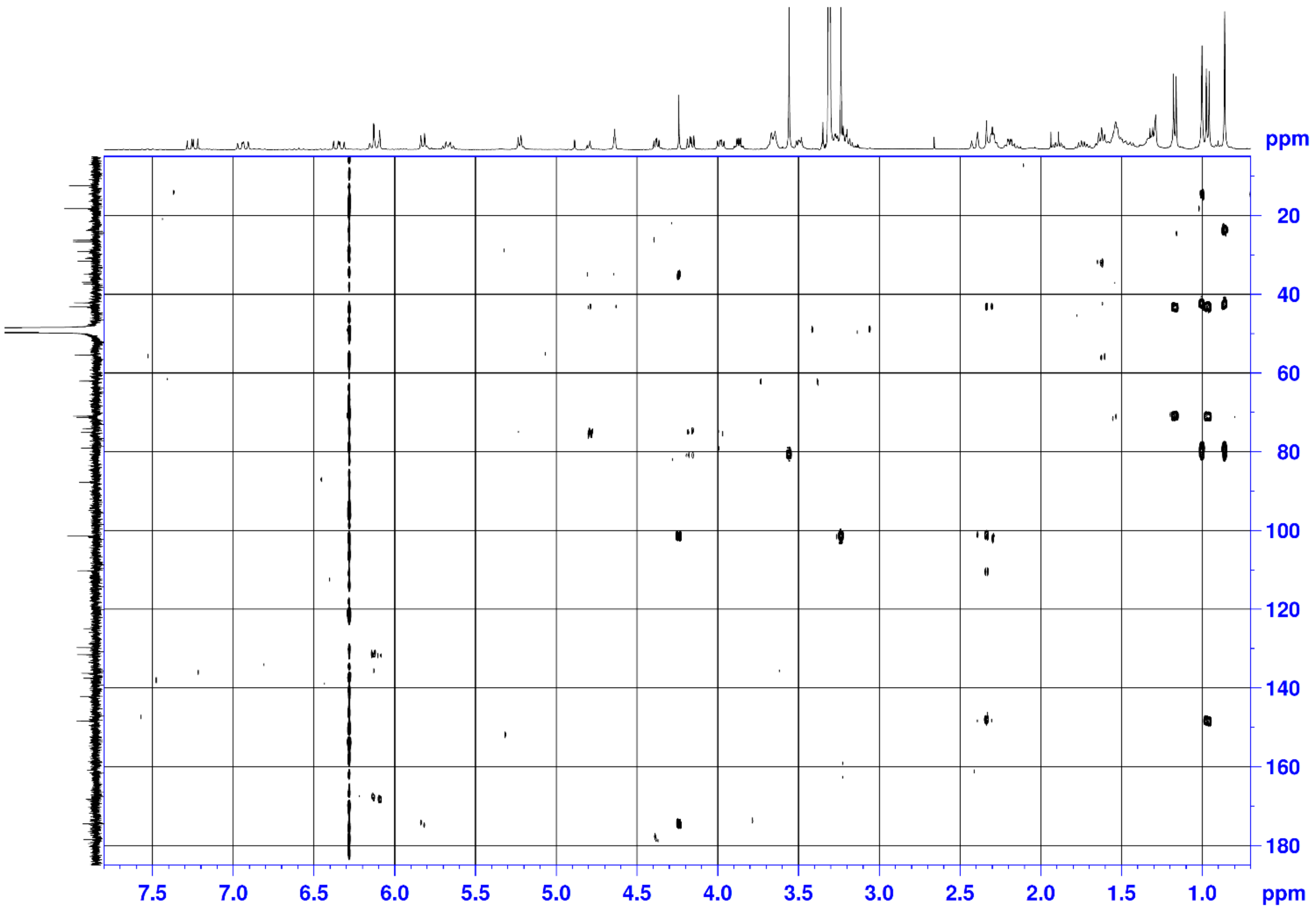

Figure S12. HMBC spectrum of 6*Z*-onnamide A (**2**) in MeOD.

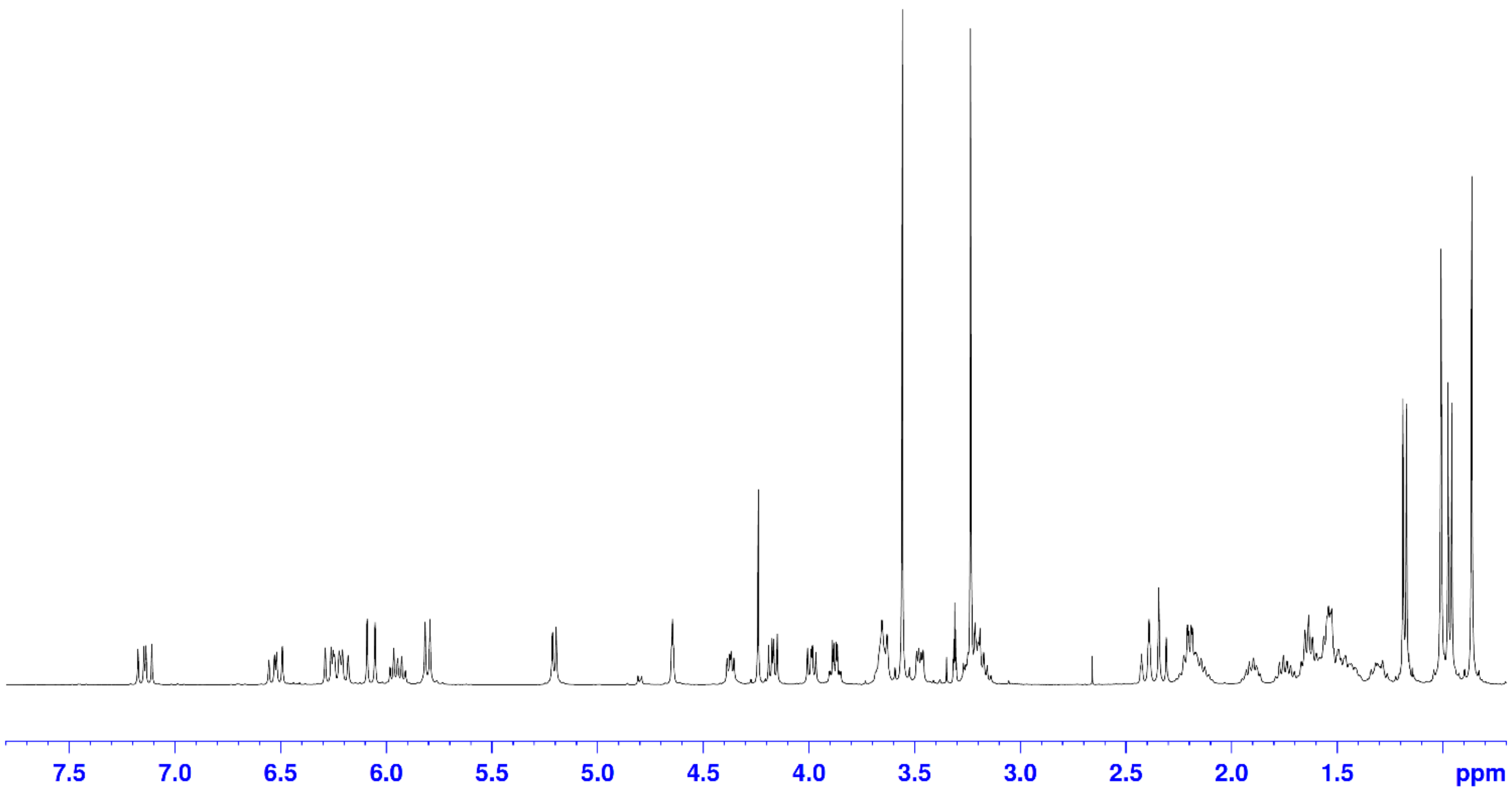

Figure S13. ^{1}H NMR spectrum of onnamide A (**3**) in MeOD.

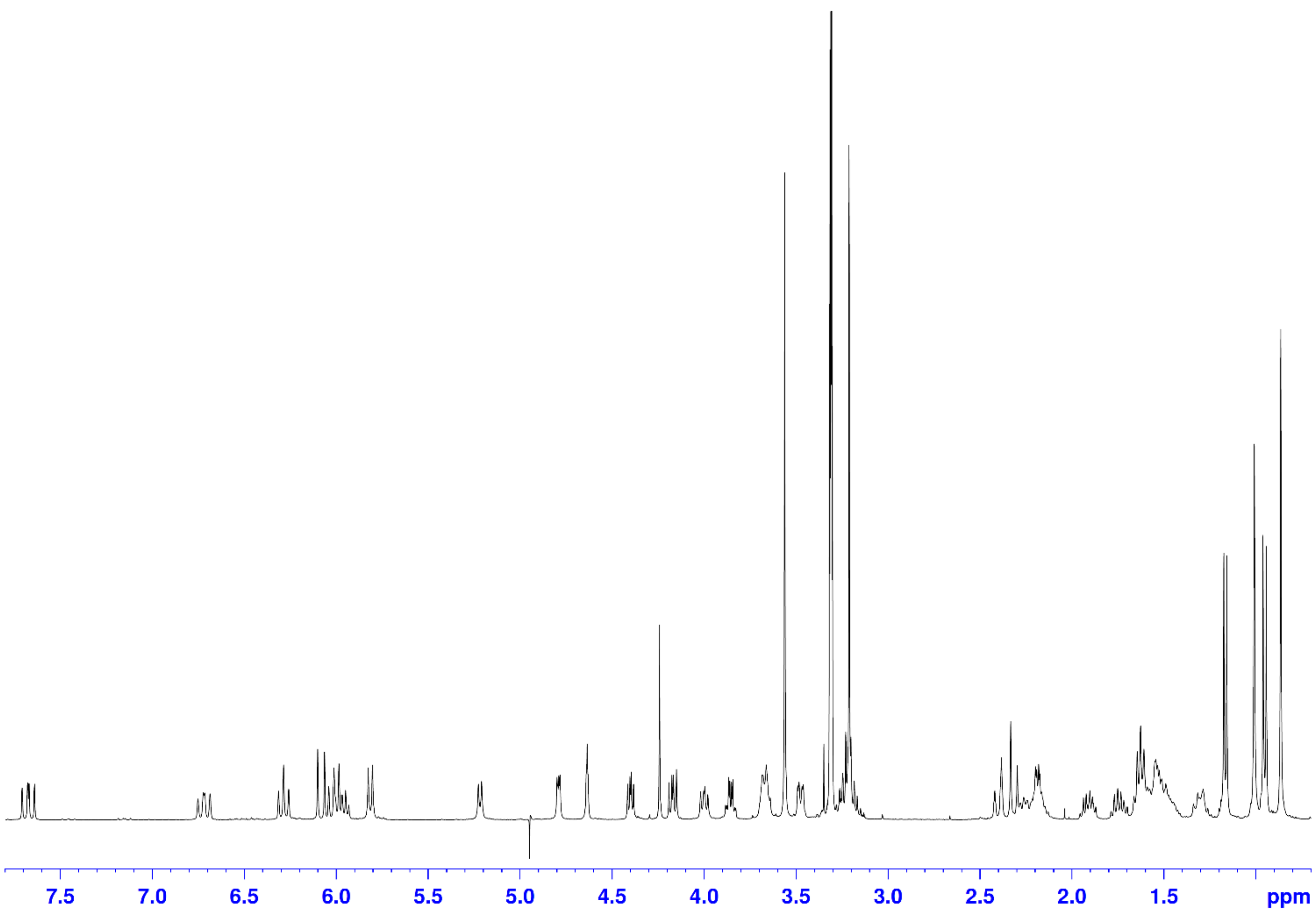

Figure S14. ^{1}H NMR spectrum of 4*Z*-onnamide A (**4**) in MeOD.

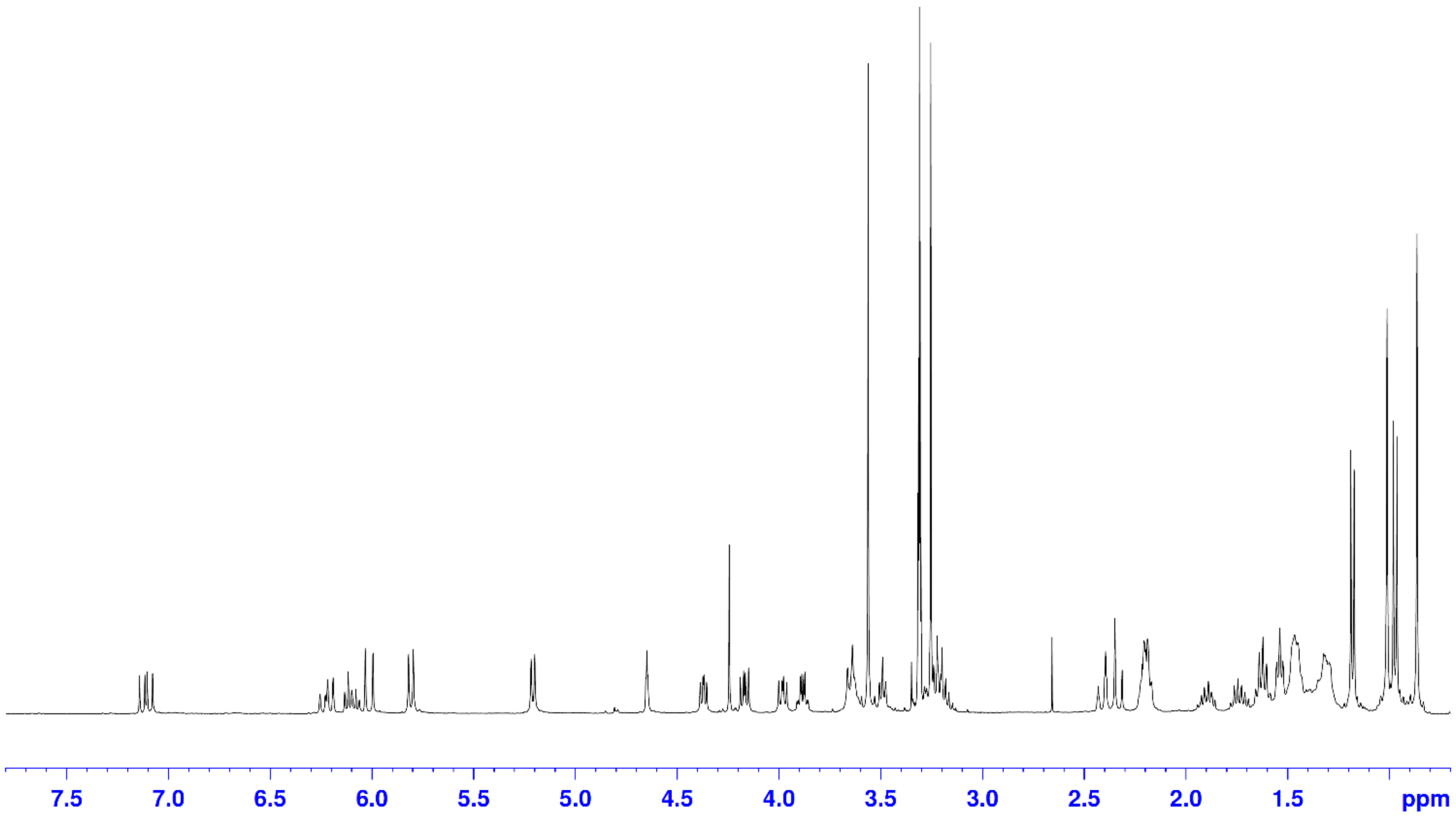

Figure S15. ^1H NMR spectrum of dihydroonnamide A (**5**) in MeOD.

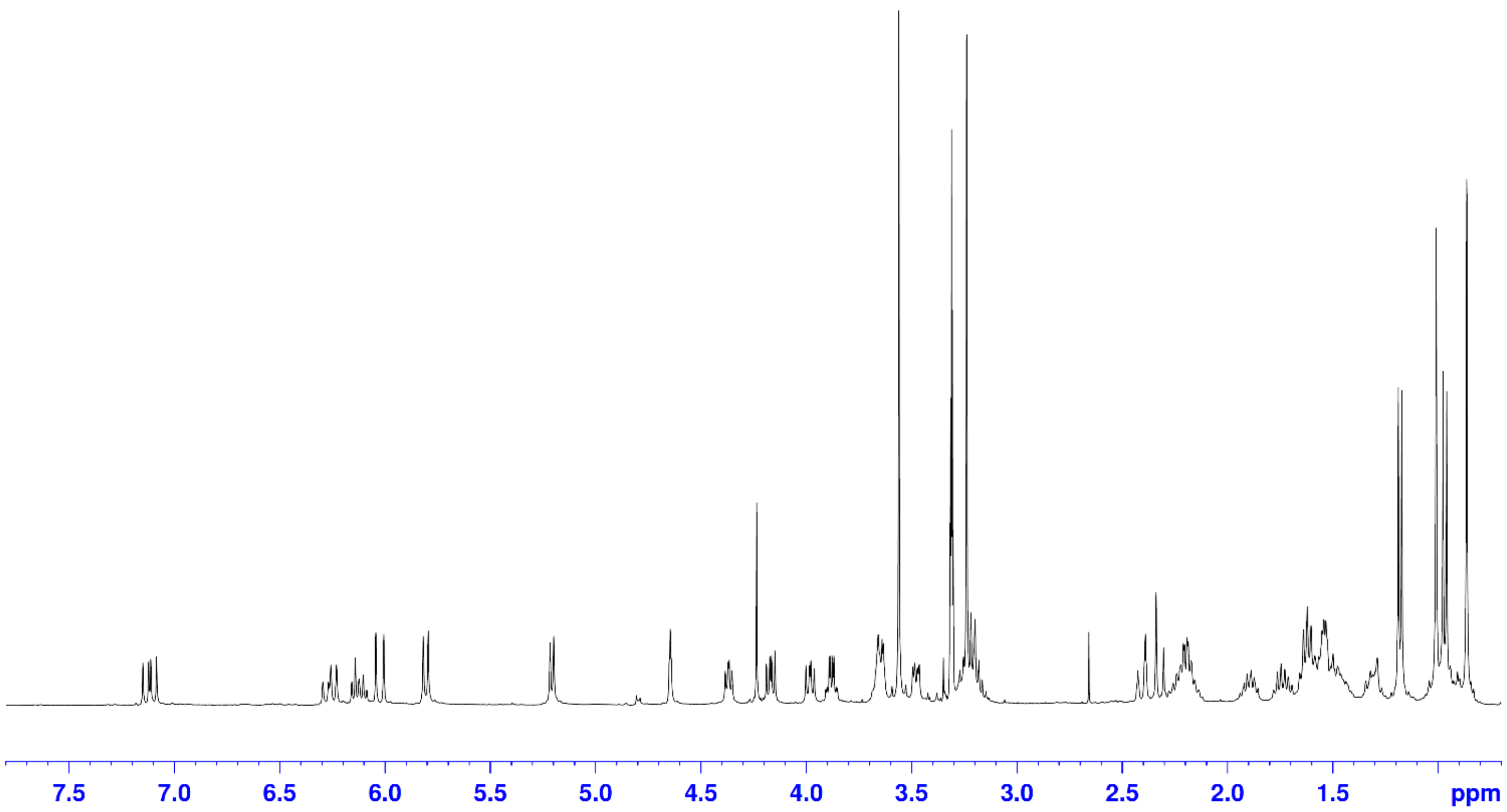

Figure S16. ^{1}H NMR spectrum of onnamide B (**6**) in MeOD.

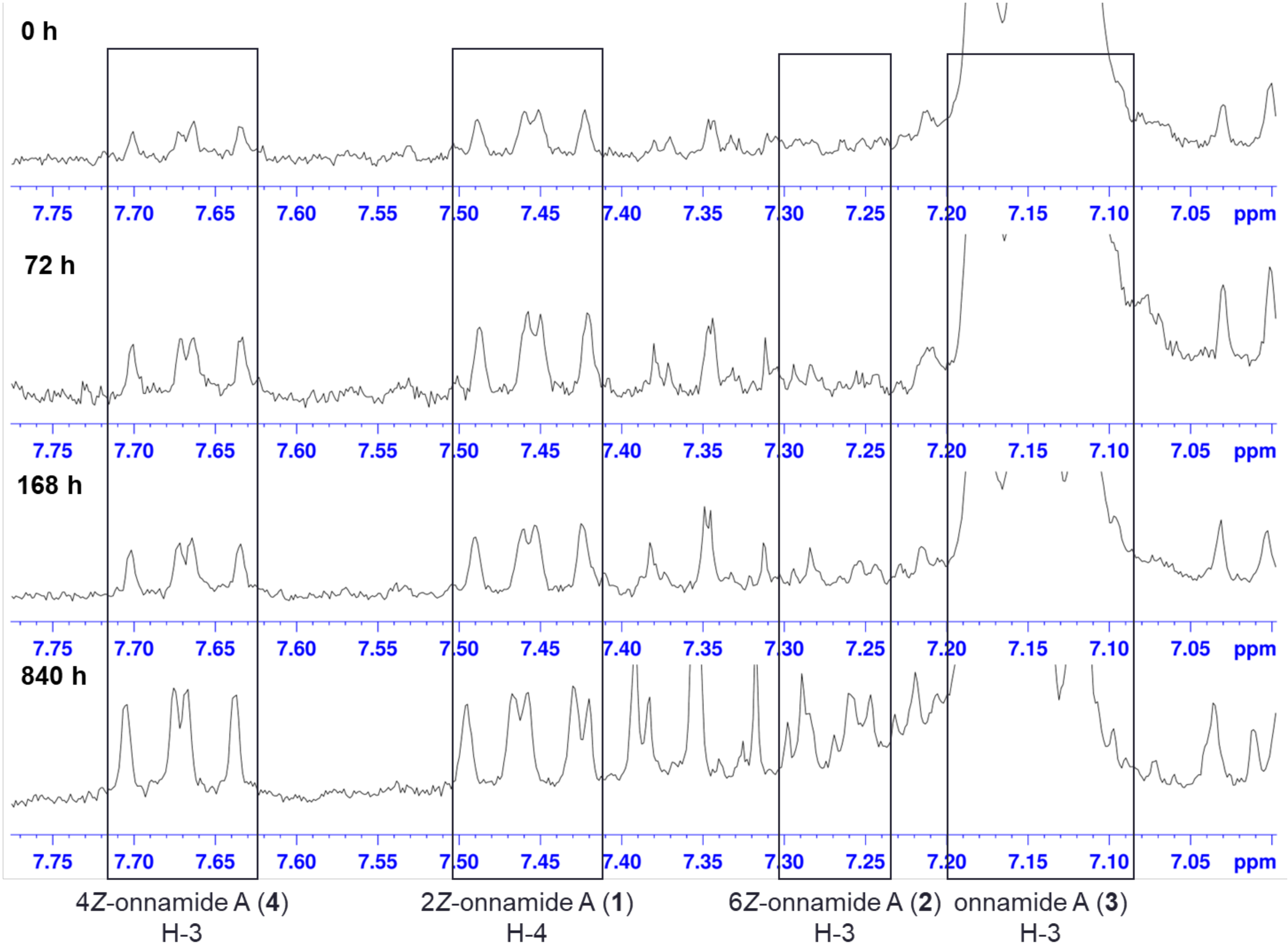

Figure S17. ^{1}H NMR spectrum of onnamide A (**3**) over time in MeOD.

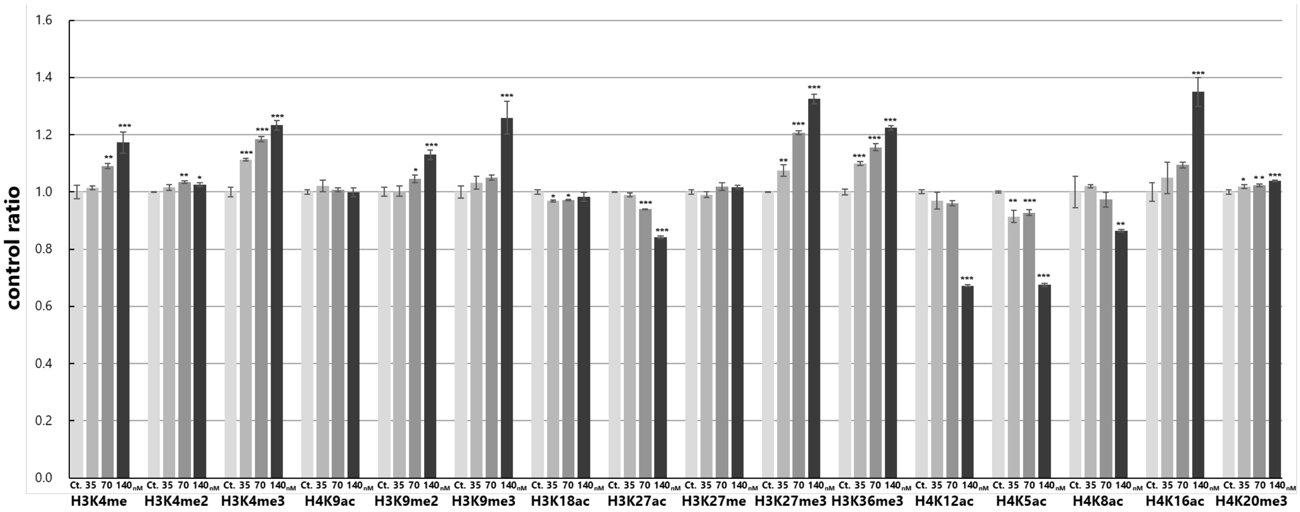

Figure S18. Effects of onnamide A (**3**) on 16 types of histone modifications. Quantification of each histone modification levels after cultivation under the medium containing each sample for 20 hours (n = 3, mean ± S.D. ***: p < 0.001, **: p < 0.01, *: p < 0.05, Dunnett test).

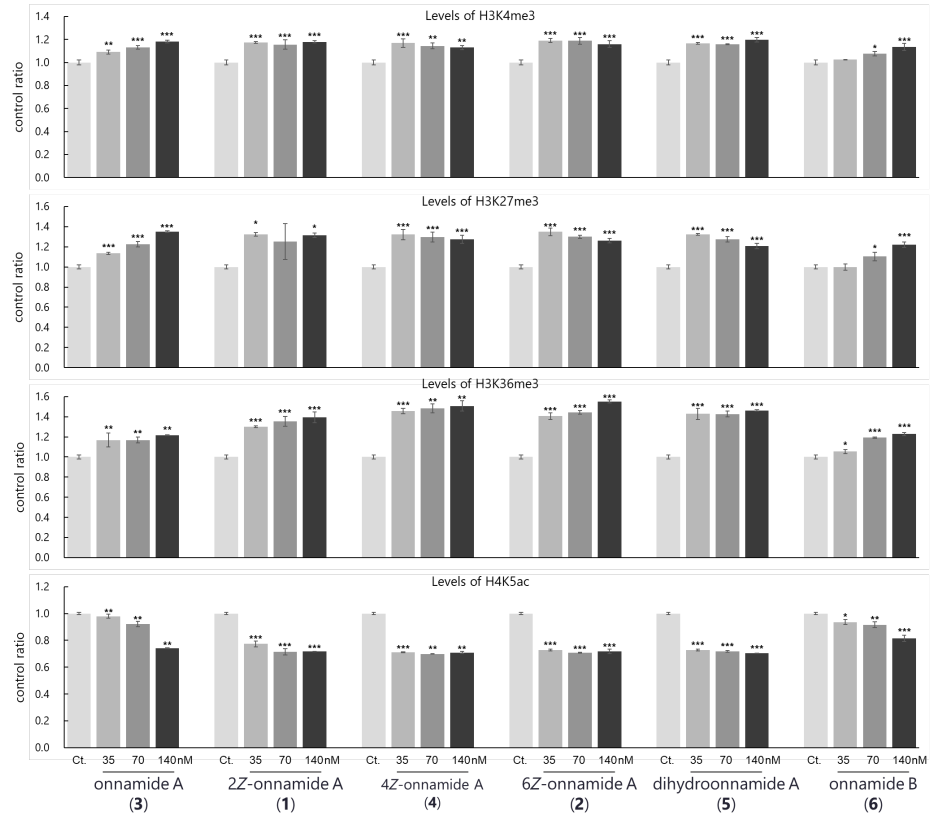

Figure S19. The levels of H3K4me3, H3K27me3, H3K36me3, and H4K5ac after onnamides (**1**-**6**)

treatment in histone modification assay (Ct.: DMSO, n = 3, mean ± S.D. ***: $p < 0.001$, **: $p < 0.01$, *: $p < 0.05$, Dunnett test).

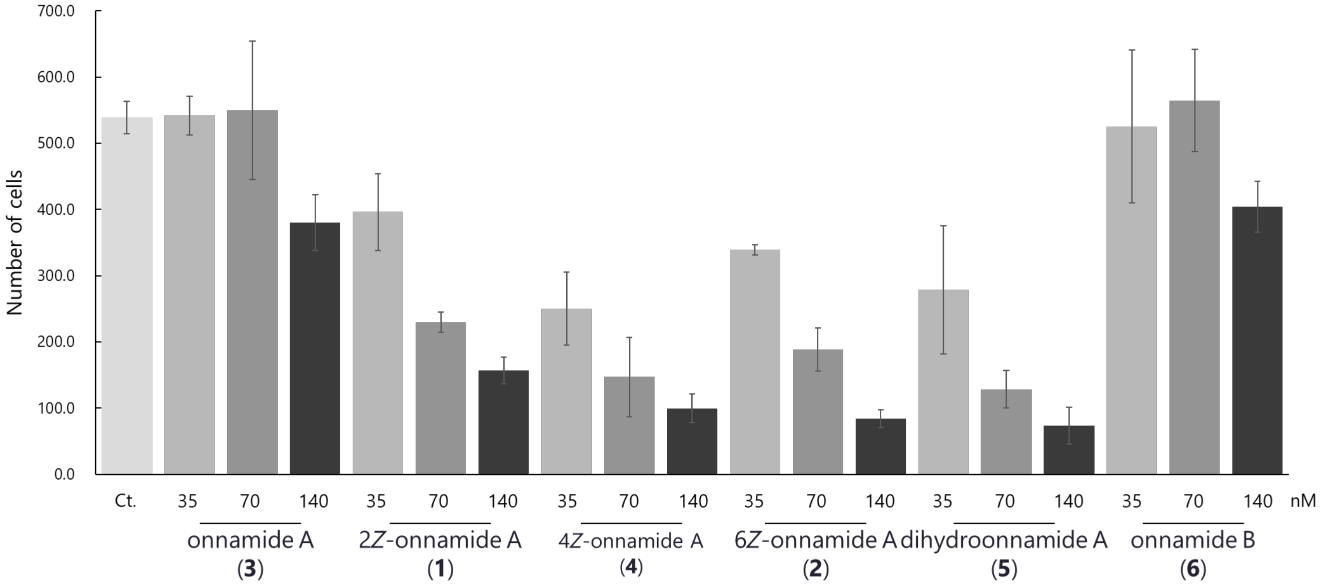

Figure S20. Effect of onnamides (**1**-**6**) treatment on number of cells in histone modification assay (Ct.: DMSO, n = 3, mean ± S.D.).

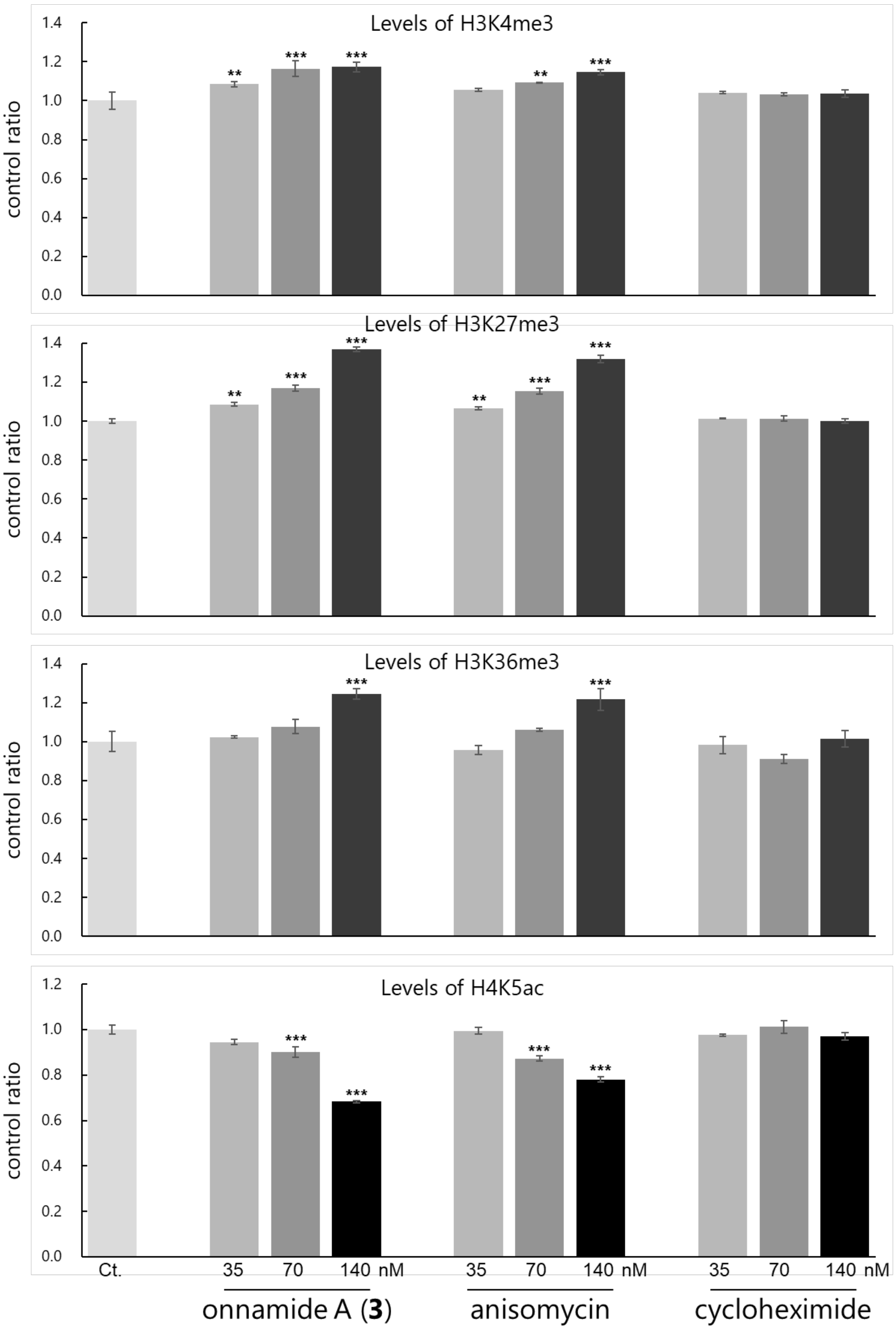

Figure S21. The levels of H3K4me3, H3K27me3, H3K36me3, and H4K5ac after onnamide A (**3**) and anisomycin treatment in histone modification assay**.** (Ct.: DMSO, n = 3, mean ± S.D. ***: $p < 0.001$, **: $p < 0.01$, Dunnett test).
